# A Clinical Introduction to the Practice of Auscultation and Other Modes of Physical Diagnosis in Diseases of the Lungs and Heart

**Published:** 1854-07

**Authors:** 


*A Clinical Introduction to the practice of Auscultation and other modes of Physical Diagnosis in Diseases of the Lungs and Heart.* By B. M. Hughes, M. D., Fellow of the Royal College of Physicians, assistant Physician to Guy's Hospital, &c. Second American, from the second and revised english Edition. Philadelphia: Blanchard and Lea. 1854.

This is a concise elementary treatise, of 304 duodecemo pages, on the subject of Auscultation and Percussion. It is designed chiefly for the use of students and such as are learning the art of Physical exploration and diagnosis. It is a plain practical Manual, well adapted for the purpose of elementary instruction.

From the hasty examination which we have given the work, we feel confident it is among the best Manuals within the reach of the Student, though it is not without omissions and defects.

For sale at Keen's Book Store, Chicago.

